# Novel CAR T-cell therapies for relapsed/refractory B-cell malignancies: latest updates from 2023 ASH annual meeting

**DOI:** 10.1186/s40164-024-00508-4

**Published:** 2024-04-18

**Authors:** Wenjie Zhang, Sumei Li, Jinlan Long, Shufeng Xie, Minghui Wang, Han Liu, Zhenshu Xu

**Affiliations:** 1 Fujian Institute of Hematology, Fujian Provincial Key Laboratory on Hematology, Fujian Medical University Union Hospital, 29 Xinquan Rd, Fuzhou, 350001 China; 2 College of Chinese Traditional Medicine, Shandong University of Traditional Chinese Medicine, Jinan, 250013 China; 3 Shanghai Institute of Hematology, State Key Laboratory of Medical Genomics, National Research Center for Translational Medicine at Shanghai, Ruijin Hospital, Shanghai Jiao Tong University School of Medicine, Shanghai, 200025 China

**Keywords:** CAR T-cell therapy, Relapsed/refractory B-cell malignancies, ASH 2023

## Abstract

Chimeric antigen receptors (CAR) are engineered fusion proteins that target T-cells to specific surface antigens of tumor cells to generate effective anti-tumor responses. CAR T-cell therapy is playing an increasingly important role in the treatment of relapsed/refractory B-cell malignancies (R/R BCM). Attempting to make CAR T-cells safer and more effective in treating R/R BCM, various novel engineered CAR T-cell agents are currently in the research and development or clinical trial stages. We have summarized here the latest reports on the novel CAR T-cell therapies for R/R BCM presented at the 2023 ASH Annual Meeting as well as the latest updates in related clinical trials.

## To the editor

CAR T-cell therapy has become an effective treatment for relapsed/refractory B-cell malignancies (R/R BCM). However, due to serious adverse events and failure to control or eradicate malignancies, many patients still cannot benefit from CAR T-cell therapy [1]. How to make CAR T-cells produce durable, effective, and safe anti-tumor effects remains a key issue. For this purpose, new targets are being studied or screened, and CAR T-cells are also being redesigned and modified [2]. Variously engineered CAR T-cells are in clinical trials. We have summarized the latest reports on novel CAR T-cell therapies for R/R BCM from the 2023 ASH Annual Meeting (ASH 2023) as well as the latest updates related to clinical trials.

## Novel modified CD19 targeted CAR T-cell therapy

CD19 CAR T-cells have always been the mainstay of CAR T-cell therapy for R/R BCM [3]. However, successful CAR T-cell therapy not only requires effective and durable clinical efficacy but also safe and tolerable toxicity [4]. At ASH 2023, several studies reported novel modified CD19 targeted CAR T-cell agents that not only ensure CAR T-cells efficacy but also alleviate adverse events such as severe cytokine release syndrome (CRS) and immune effector cell-associated neurotoxicity syndrome (ICANS). Kang et al. reported novel CD19 targeted CAR T-cells incorporating a small hairpin RNA element to silence the interleukin-6 gene (ssCART-19), reducing the incidence of severe CRS and neurotoxicity in R/R B-cell acute lymphoblastic leukemia patients [5]. Compared with the control group the incidence of grade 3–4 CRS in the ssCART-19 group decreased by 22.61% and there was no occurrence of grade 3–4 ICANS. The complete response (CR) or CR with incomplete hematological recovery (CRi) rate of ssCART-19 treatment on day 28 was 91.49%. Another Phase 1 trial on a novel third generation CD19 directed CAR T-cells incorporating CD28 and Toll-like receptor 2 co-stimulatory domains mitigated the risk of CAR T-cell therapy related ICANS while maintaining therapeutic efficacy in R/R B-cell non-Hodgkin’s lymphoma (NHL) [6]. There was no grade ≥ 3 CRS or any grade ICANS during the treatment period, and there was still a 52% CR at month three. Park et al. created a novel CD19 CAR construct with calibrated CAR activation potential by mutating 2 out of 3 immunoreceptor tyrosine-based activation motifs, called 19(T2)28z1XX [7]. Patients with R/R diffuse large B-cell lymphomas (DLBCL) receiving 19(T2)28z1XX CAR T-cell therapy showed persistent remission, with an overall CR rate of 71%, and a low incidence of severe CRS (4%) and ICANS (7%). Zheng et al. reported the latest clinical data of non-viral programmed cell death protein-1 locus specifically integrated anti-CD19 CAR T-cells (BRL-201) generated using CRISPR-Cas9 for the treatment of R/R NHL, with a median follow-up period of 29 months [8]. BRL-201 provided a safe and effective clinical response with an objective response rate (ORR) of 100% and a CR rate of 85.7%, while no grade 3–4 CRS or ICANS were observed.

## Novel CAR T-cell therapy targeting ROR-1, CD70 and dual antigens

Several studies reported the latest clinical trial data on CAR T-cells engineered to recognize new targets for the treatment of R/R BCM. Abramson et al. reported the preliminary results of UCART20×22, a dual allogeneic CAR T-cell product targeting CD20 and CD22 in R/R NHL [9]. All three enrolled patients responded with two CRs and one PR on day 28. Meanwhile, UCART20×22 exhibited good safety and acceptable toxicity. Tu et al. published the results of CD19/CD70 CAR T-cell therapy for R/R DLBCL [10]. The CR rate at 1 month reached 75.0%. After a median follow-up time of 19.9 months, 50% of patients still maintained CR, with a median disease-free survival of 10.5 months. No patients experienced a grade ≥ 3 CRS or any grade ICANS. In addition, a bispecific anti-CD20/CD19 CAR T-cell agent, C-CAR039, demonstrated durable responses and good safety in R/R B-NHL therapy [11]. The ORR and CR rates were 91.5% and 85.1%. Meanwhile, only one patient (2.1%) experienced severe CRS and there was no occurrence of severe ICANS. Another ongoing phase 1/2 trial is evaluating the effectiveness and safety of ROR1-specific CAR T-cell therapy for R/R aggressive B-cell lymphoma, including LBCL and Mantle cell lymphoma [12]. This study consists of two stages: phase 1 is for dose escalation while phase 2 is for dose expansion.

In conclusion, ASH 2023 presented the prospects and progress of novel CAR T-cell therapies for the treatment of R/R BCM as summarized in Tables 1 and 2. At present, the failure of CAR T-cell therapy is usually attributed to the loss of target antigens or depletion of CAR T-cells, while dual antigen targeting and CAR T-cells modification are potential strategies to overcome treatment failure. Therefore, the emergence of novel CAR T-cell therapies will further improve the treatment safety and effectiveness of patients with R/R BCM.

**Table 1:** Novel CAR T-cell agents for relapsed/refractory B-cell malignancies

Product	Phase	Target	Reconstruction method	Indication	T cells	Clinical trial identifier	References
ssCART−19	I/II	CD19	shRNA-IL−6 gene silencing element	R/R B-ALL	Autologous	NCT03919240	[5]
WZTL−002	I	CD19	CD28 and TLR2 co-stimulatory domains	R/R B-NHL	Autologous	NCT04049513	[6]
19(T2)28z1XX	I	CD19	Mutating 2 of the 3 ITAMs	R/R DLBCL	Autologous	NCT04464200	[7]
BRL−201	I	CD19	Non-viral PD−1 locus specific integration via CRISPR-Cas9	R/R B-NHL	Autologous	NCT04213469	[8]
UCART20×22	I/IIa	CD20/CD22	N/A	R/R B-NHL	Donor	NCT05607420	[9]
CD19/CD70 CAR T-cells	I/II	CD19/CD70	N/A	R/R DLBCL	Autologous	NCT03125577	[10]
C-CAR039	I	CD20/CD19	N/A	R/R B-NHL	Autologous	NCT04693676/NCT04696432/NCT04655677/NCT04317885	[11]
ONCT−808	I/II	ROR1	N/A	R/R BCL	Autologous	NCT05588440	[12]

R/R: Relapsed/refractory, B-ALL: B-cell acute lymphoblastic leukemia, B-NHL: B-cell non-Hodgkin’s lymphoma, DLBCL: Diffuse large B-cell lymphomas, BCL: B-cell Lymphoma, IL−6: Interleukin−6, TLR2: Toll-like receptor 2, ITAMs: Immunoreceptor tyrosine-based activation motifs, PD−1: Programmed cell death protein−1

**Table 2:** The efficacy and safety of novel CAR T-cells

Product	Patients	Conditioning regimen	CRS	ICANS	ORR/CR/CRi	PFS	OS	References
ssCART−19	47	Fludarabine at 30mg/m^2^/day, cyclophosphamide at 300mg/m^2^/day on D−5,−4, and−3	Grade 1–2 25/47 (53.20%) Grade 3–4 7/47 (14.89%)	Grade 1 2/47 (4.26%)	91.49% CR/CRi on day 28	mPFS 14.7 months	N/A	[5]
WZTL−002	21	Fludarabine (30 mg/m^2^/day) and cyclophosphamide (500mg/m^2^/day) on D−5,−4, and−3	Grade 1–2 13/21 (62%)	No ICANS of any grade	52% CR at 3 months	N/A	N/A	[6]
19(T2)28z1XX	28	Fludarabine and cyclophosphamide	Grade 1–2 24/28 (86%) Grade 3 1/28 (4%)	Grade 1–2 1/28 (4%) Grade 3 2/28 (7%)	82% ORR 71% CR	N/A	N/A	[7]
BRL−201	21	Cyclophosphamide (500mg/m^2^, D−3 to−2) and fludarabine (30mg/m^2^, D−4 to−2)	Grade 1–2 14/21 (66.7%)	Grade 1–2 4/21 (19.0%)	100% ORR 85.7% CR	mPFS 20.8 months	76.2% 12-month OS	[8]
UCART20×22	3	Fludarabine 30 mg/m^2^ × 3d, cyclophosphamide 0.5 g/m^2^ × 3d, alemtuzumab 12 mg on D1, 24 mg on D2, D3	Grade 1–2 3/3 (100%)	No ICANS of any grade	66.7% CR on day 28	N/A	N/A	[9]
CD19/CD70 CAR T-cells	8	Cyclophosphamide and fludarabine chemotherapy conditioning 1–2 days	Grade 1–2 3/8 (37.5%)	No ICANS of any grade	87.5% ORR 75.0% CR at 1 month	N/A	N/A	[10]
C-CAR039	48	Cyclophosphamide plus fludarabine chemotherapy conditioning 3 days	Grade 1–2 44/48 (91.7%) Grade 3 1/48 (2.1%)	Grade 1–2 3/48 (6.52%)	91.5% ORR 85.1% CR	66.0% 24-month PFS	77.9% 24-month OS	[11]
ONCT−808	N/A	Cyclophosphamide and fludarabine	N/A	N/A	N/A	N/A	N/A	[12]

CRS: Severe cytokine release syndrome, ICANS: Immune effector cell-associated neurotoxicity syndrome, ORR: Overall response rate, CR: Complete response, CRi: CR with incomplete hematological recovery, mPFS: Median progression free survival, OS: Overall survival

## Data Availability

The material supporting the conclusion of this study has been included within the article.

## Abbreviations

ASH: American Society of Hematology
CAR: Chimeric antigen receptor
R/R: Relapsed/refractory
BCM: B-cell malignancies
DLBCL: Diffuse large B-cell lymphomas
NHL: Non-Hodgkin’s lymphoma
CRS: Cytokine release syndrome
ICANS: Immune effector cell-associated neurotoxicity syndrome
CR: Complete response
CRi: CR with incomplete hematological recovery
ORR: Objective response rate
PFS: Progression free survival
OS: Overall survival
